# Current practice of diagnosis and treatment for rectourethral fistula in male patients with anorectal malformation: a multicenter questionnaire survey in Japan

**DOI:** 10.1007/s00383-024-05801-1

**Published:** 2024-08-09

**Authors:** Toshio Harumatsu, Masakazu Murakami, Koshiro Sugita, Tetsuya Ishimaru, Akihiro Fujino, Mitsuyuki Nakata, Shigeyoshi Aoi, Hideki Soh, Yoshiaki Kinoshita, Keiichi Uchida, Takeshi Hirabayashi, Yasushi Fuchimoto, Hideaki Okajima, Takeo Yonekura, Tsugumichi Koshinaga, Minoru Yagi, Hiroshi Matsufuji, Seiichi Hirobe, Masaki Nio, Shigeru Ueno, Jun Iwai, Tatsuo Kuroda, Satoshi Ieiri

**Affiliations:** 1 Department of Pediatric Surgery, Medical and Dental Sciences, Research and Education Assembly, Research Field in Medicine and Health Sciences, Kagoshima University, Kagoshima, Japan; 2 Division of Pediatric Surgery, National Center for Child Health and Development, Tokyo, Japan; 3 Japanese Study Group of Anorectal Anomalies, Tokyo, Japan; 4 Department of Pediatric Surgery, School of Medicine, Keio University, Tokyo, Japan; 5 Department of Pediatric Surgery, Chiba Children’s Hospital, Chiba, Japan; 6 Department of Pediatric Surgery, Kyoto Prefectural University of Medicine, Kyoto, Japan; 7 Department of Pediatric Surgery, Kawasaki Medical School, Kurashiki, Japan; 8 Department of Pediatric Surgery, Niigata University Graduate School, Niigata, Japan; 9 Department of Pediatric Surgery, Mie Prefectural General Medical Center, Tsu, Japan; 10 Pediatric Surgery, Hirosaki University Hospital, Hirosaki, Japan; 11 Department of Pediatric Surgery, International University of Health and Welfare, Narita, Japan; 12 Department of Pediatric Surgery, Kanazawa Medical University, Kanazawa, Japan; 13 Department of Pediatric Surgery, Nara Prefectural General Medical Center, Nara, Japan; 14 Department of Pediatric Surgery, Nihon University School of Medicine, Tokyo, Japan; 15 Shonai Hospital, Tsuruoka City, Shonai, Japan; 16 St. Luke International Hospital, Tokyo, Japan; 17 Tokyo Metropolitan Children’s Medical Center, Tokyo, Japan; 18 Tohoku Kosai Hospital, Sendai, Japan; 19 Okamura Isshin-do Hospital, Okayama, Japan; 20 Kanagawa Children’s Medical Center, Yokohama, Japan

**Keywords:** Anorectal malformation, Male sex, Anorectoplasty, Posterior sagittal anorectoplasty, Laparoscopy-assisted anorectoplasty, Multicenter survey

## Abstract

**Purpose:**

Surgical procedures for anorectoplasty for anorectal malformations (ARMs), particularly rectourethral fistula (RUF), depend on the institution. We investigated the diagnosis and treatment of RUF in male patients with ARMs in Japan using a questionnaire survey.

**Methods:**

An online survey inquiring about the diagnosis and treatment (diagnostic modalities, surgical approaches, fistula dissection devices, and fistula closure techniques) of each type of ARM in male patients was conducted among institutional members of the Japanese Study Group of Anorectal Anomalies. Fisher’s exact test was used to compare surgical methods between posterior sagittal anorectoplasty (PSARP) and laparoscopy-assisted anorectoplasty (LAARP).

**Results:**

Sixty-one institutions (100%) completed the survey. LAARP was the preferred approach for high-type ARM (75.4%). PSARP was preferred for intermediate-type ARM (59.0%). Monopolar devices were most commonly used (72.1%) for RUF dissection. Blunt dissection was more frequent in the PSARP group (PSARP vs. LAARP: 55.6 vs. 20.0%, *p* < 0.005). Cystoscopy/urethroscopy to confirm the extent of dissection was used more frequently in the LAARP group (70.0% vs. 25.0%, *p* < 0.005). Clips and staplers were used more frequently in the LAARP group (*p* < 0.05).

**Conclusion:**

Distinct fistula management strategies for PSARP and LAARP were revealed. Further studies are needed to investigate the postoperative outcomes associated with these practices.

## Introduction

Anorectal malformations (ARMs) represent a spectrum of congenital anomalies, affecting 1 in 4000–5000 live births [1]. Despite advances in surgical techniques, poor functional outcomes remain a major concern, especially in male patients with recto-vesical/recto-prostatic fistulas [1]. Laparoscopy-assisted anorectoplasty (LAARP), introduced in 2000 [2], has gained popularity over the last 2 decades as an alternative to posterior sagittal anorectoplasty (PSARP).

Several studies have compared the outcomes of LAARP and PSARP, highlighting the advantages and limitations of each approach [3, 4]. LAARP offers improved visualization, minimal perineal dissection, and accurate fistula placement [2]. However, reports of urethral diverticula and residual fistulas after LAARP have raised severe postoperative problems, including large posterior urethral diverticula and residual fistulas that can cause urinary incontinence, infection, stone formation, and even malignant transformation long after the procedure [5, 6].

No nationwide surveys from Japan have provided detailed information on surgical techniques and associated fistula treatment strategies for ARMs.

Through a questionnaire survey, the present study investigated the technical aspects and strategies employed in the diagnosis and treatment of rectourethral fistulas (RUF) of anorectoplasty for male patients with intermediate and high types of ARMs.

## Materials and methods

### Study participants

The participants in this survey were 61 institutional members associated with the Japan Society of ARM Study Group, which is a leading organization in the field of ARMs in Japan.

### Survey questionnaire

The questionnaire was devised by researchers at Kagoshima University and evaluated and revised by the scientific committee of the Japanese Study Group of Anorectal Anomalies. The survey inquired about the evaluation and treatment of each type of ARM in male patients, including diagnostic modalities, surgical approaches, fistula dissection devices, and fistula closure techniques.

The diagnosis of the type of ARMs was obtained by confirming blind-end positions and fistula locations using imaging modalities such as invertograms, ultrasonography, contrast studies, magnetic resonance imaging (MRI), and endoscopy. We also asked about the timing and procedure of definitive surgery performed for anorectoplasty in different types of malformations, the use of laparoscopy, devices utilized for fistula dissection, techniques for confirming the dissection extent and closing fistulas on the rectal/urethral side, as well as the materials employed for fistula closure and the appropriate sites for separating fistulas.

### Survey procedures

An anonymous online survey was created using www.surveymonkey.com (Survey Monkey, Portland, OR, USA). The survey was distributed by email with a link to the online survey before the annual meeting of the Japan Society of ARM Study Group in 2023. The survey was open for 6 months, and one email reminder was sent during this period.

### Statistical analyses

Fisher’s exact test was used for subgroup analysis to compare the surgical techniques and methods used for RUF treatment between the groups that underwent PSARP and LAARP for the treatment of intermediate-type ARM. Odds ratios (ORs) and 95% confidence intervals (CIs) were calculated to assess relative differences. Statistical significance was set than 0.05. All statistical analyses were performed using EZR (Saitama Medical Center, Jichi Medical University, Saitama, Japan), a graphical user interface for R (The R Foundation for Statistical Computing, Vienna, Austria). More precisely, it is a modified version of R commander designed to add statistical functions frequently used in biostatistics [7].

### Ethical approval

The institutional Review Board decided that there was no need for ethical approval because this study was a questionnaire survey focusing on the technical aspects of diagnosis and treatment of ARMs and did not involve any interventions or collection of personal information of patients.

The study design and questionnaire were approved by the Scientific Committee of the Japan Society of the ARM Study Group. All 61 institutions received an explanation of the study objective, and informed consent was obtained from them during the study. The survey and collected data were anonymous, thus maintaining the privacy of the participants. Participants had the right to retract their responses at any time. The datasets generated and/or analyzed during the current study are available from the corresponding author upon reasonable request.

## Results

### Methods for assessing the types of ARM in male patients

The results of the questionnaire regarding the diagnostic methods for assessing the types of ARM in male patients are presented in Table 1. All 61 institutions (100%) responded to the questionnaire.

**Table 1:** Diagnostic methods for assessing the types of ARM in male patients

	Number of institutions (%)
What methods do you use to verify the location of the rectal end after birth? (some overlapping)	
Invertogram	53/61 (86.9%)
Prone cross-table	16/61 (26.2%)
Ultrasonography	40/61 (65.6%)
What methods do you use to evaluate rectourethral fistulas? (some overlapping)	
Distal colostogram	61/61 (100%)
MRI	13/61 (21.3%)
CT	2/61 (3.3%)
Ultrasonography	15/61 (24.6%)
Cystoscope/urethroscopy	31/61 (50.8%)
Total, *n* (%)	61 (100%)

*ARM* anorectal malformation, *MRI* magnetic resonance imaging, *CT* computed tomography

Regarding the diagnostic methods used to verify the location of the rectal end after birth, 53 (86.9%) institutions used invertography, 16 (26.2%) used prone cross-table radiography, and 40 (65.6%) used ultrasonography.

For the evaluation of rectourethral fistulas, all 61 (100%) institutions used distal colostograms, 13 (21.3%) used MRI, 2 (3.3%) used computed tomography (CT), 15 (24.6%) used ultrasonography, and 31 (50.8%) used cystoscopy or urethroscopy.

### Preferred age for anorectoplasty in male patients with ARM without severe associated anomalies

**Fig. 1:**
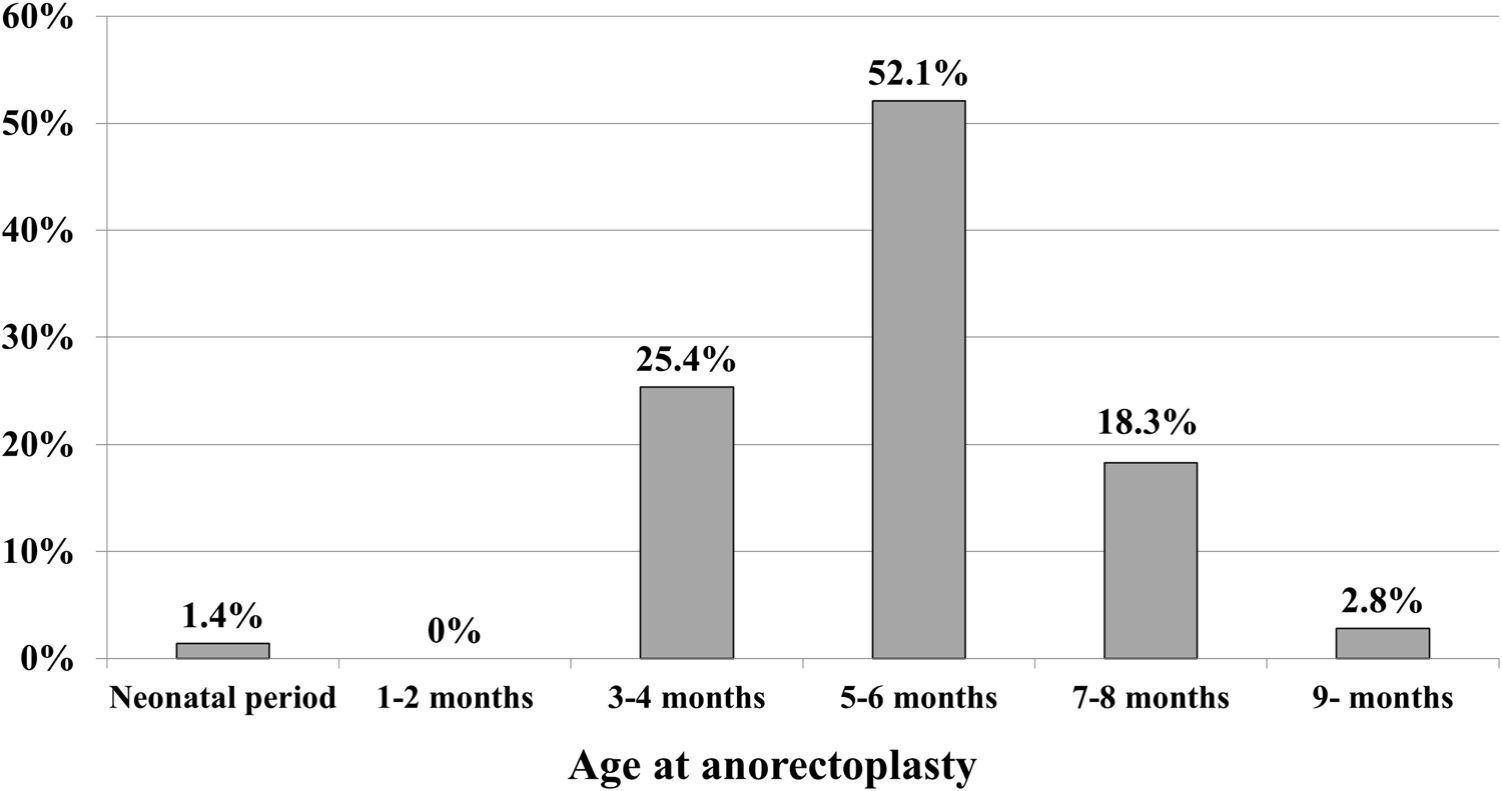
Preferred age at anorectoplasty in ARM cases without severe associated anomalies. ARM, anorectal malformation

Figure 1 shows the results of the preferred age for anorectoplasty in male patients with ARMs without severe associated anomalies such as congenital heart disease, esophageal atresia, or chromosomal anomalies, which may affect the timing of anorectoplasty. Most institutions (52.1%) performed definitive surgery at 5–6 months of age, followed by 25.4% at 3–4 months, 18.3% at 7–8 months, and 2.8% at 9 months or later. Only 1.4% of institutions performed surgery during the neonatal period, and none reported performing definitive surgery at 1–2 months of age.

### Surgical procedures for high-/ intermediate-type ARM and the range of laparoscopy usage

Table 2 summarizes the results of the questionnaire on surgical procedures for high-/intermediate-type ARM and the range of laparoscopy usage. For high-type ARM, 46 institutions (75.4%) performed LAARP, 13 institutions (21.3%) performed PSARP, 6 institutions (9.8%) performed sacroperineal or sacroabdominoperineal anorectoplasty (SP-SAP), and 1 institution (1.6%) performed anterior sagittal anorectoplasty (ASARP). For intermediate-type ARM, 36 institutions (59.0%) performed PSARP, 20 institutions (32.8%) performed LAARP, 9 institutions (14.8%) performed SP-SAP, and 1 institution (1.6%) performed ASARP.

**Table 2:** Surgical procedures employed for high-/intermediate-type of ARM and the range of laparoscopy usage

What surgical procedure do you perform for high/intermediate type of ARM?	High type (some overlapping)	Intermediate type (some overlapping)
PSARP	13/61 (21.3%)	36/61 (59.0%)
LAARP	46/61 (75.4%)	20/61 (32.8%)
SP-SAP	6/61 (9.8%)	9/61 (14.8%)
ASARP	1/61 (1.6%)	1/61 (1.6%)

What procedure do you use laparoscopy?	PSARP (*n* = 13) (some overlapping)	LAARP (*n* = 46) (some overlapping)	PSARP (*n* = 36) (some overlapping)	LAARP (*n* = 20) (some overlapping)
Entire operative procedure (complete laparoscopic surgery)	0/13 (0%)	37/46 (80.4%)	0/36 (0%)	15/20 (75.0%)
Only for dissection of the fistula	3/13 (23.1%)	9/46 (19.6%)	2/36 (5.6%)	5/20 (15.0%)
No usage for relatively low cases among high-/intermediate type	7/13 (53.8%)	5/46 (10.9%)	5/36 (13.9%)	2/20 (10.0%)
No usage	4/13 (30.8%)	0/46 (0%)	28/36 (77.8%)	0/20(0%)

*ARM* anorectal malformation, *PSARP* posterior sagittal anorectoplasty, *LAARP* laparoscopy-assisted anorectoplasty, *SP-SAP* sacroperineal or sacroabdominoperineal anorectoplasty, *ASARP* Anterior sagittal anorectoplasty

A subgroup analysis was also performed to evaluate the range of laparoscopy use in PSARP and LAARP for both high- and intermediate-type ARM. In high-type ARM, 37 institutions (80.4%) used laparoscopy for the entire operative procedure in LAARP, while nine institutions (19.6%) used it only for dissection of the fistula. In PSARP for high-type ARM, seven institutions (53.8%) did not use laparoscopy for relatively low cases, four (30.8%) did not use it at all, and three institutions (23.1%) used it only for dissection of the fistula.

For intermediate-type ARM, 15 institutions (75.0%) used laparoscopy for the entire operative procedure in LAARP, 5 institutions (15.0%) used it only for dissection of the fistula, and 2 institutions (10.0%) did not use it for relatively low cases. In the PSARP for intermediate-type ARM, 28 institutions (77.8%) did not use laparoscopy at all, five institutions (13.9%) did not use it for relatively low cases, and two institutions (5.6%) used it only for dissection of the fistula.

### The methods of dissection for RUF in all ARMs and subgroup analysis in intermediate type of ARMs

Table 3 summarizes the results of the questionnaire on the methods of rectourethral fistula dissection. Regarding the main device used to dissect the rectourethral fistula in all ARMs, 44 institutions (72.1%) used monopolar devices such as hooks, spatula, or scissors, 14 institutions (23.0%) used bipolar devices such as scissors, 14 institutions (23.0%) used ultrasonically activated devices (USAD), 11 institutions (18.0%) used scissors, and 31 institutions (50.8%) used blunt dissection.

**Table 3:** The methods of dissection for RUF in all ARMs and subgroup analysis in intermediate type of ARMs

	All ARMs (some overlapping)	Subgroup analysis in intermediate type of ARMs				
		PSARP (*n* = 36) (some overlapping)	LAARP (*n* = 20) (some overlapping)	OR	95% CI	*p* value
What is the main device used for dissecting the rectourethral fistula? (some overlapping)						
Monopolar (e.g., hook, spatula, and scissors)	44/61 (72.1%)	30/36 (83.3%)	14/20 (70.0%)	2.11	0.47–9.55	0.31
Bipolar (e.g., scissors)	14/61 (23.0%)	8/36 (22.2%)	7/20 (35.0%)	0.54	0.14–2.15	0.35
Ultrasonically activated device (USAD)	14/61 (23.0%)	9/36 (25.0%)	8/20 (40.0%)	0.51	0.13–1.91	0.36
Scissors	11/61 (18.0%)	5/36 (13.9%)	1/20 (5.0%)	3.01	0.30–152.33	0.40
Blunt dissection	31/61 (50.8%)	20/36 (55.6%)	4/20 (20.0%)	7.63	1.83–40.1	< 0.005
How do you confirm sufficient dissection of the rectourethral fistula? (some overlapping)						
Colonoscopy from the colostomy	5/61 (8.2%)	4/36 (11.1%)	0/20 (0%)	Inf	0.37-Inf	0.29
Cystoscopy/urethroscopy	25/61 (41.0%)	9/36 (25.0%)	14/20 (70.0%)	0.17	0.043–0.62	< 0.005
Visually confirmation by opening the rectum	23/61 (37.7%)	19/36 (52.8%)	0/20 (0%)	Inf	4.45-Inf	< 0.001
Insertion of a catheter by opening the rectum	11/61 (18.0%)	8/36 (22.2%)	2/20 (10.0%)	2.53	0.43–27.15	0.30
Only external appearance without opening the rectum	29/61 (47.5%)	15/36 (41.7%)	12/20 (60.0%)	0.48	0.13–1.66	0.27
Fluorescent ureteral catheter	3/61 (4.9%)	1/36 (2.8%)	2/20 (10.0%)	0.26	0.0042–5.39	0.29

*RUF* rectourethral fistula, *ARM* anorectal malformation, *PSARP* posterior sagittal anorectoplasty; *LAARP* laparoscopically assisted anorectoplasty, *OR* odds ratio; CI, confidence interval

Regarding the subgroup analysis in intermediate type of ARMs, blunt dissection was significantly more frequently used in the PSARP group (55.6%) than in the LAARP group (20.0%) (OR, 7.63; 95% CI 1.83–40.1, *p* < 0.005).

Regarding the confirmation the extent of dissection in all ARMs, 5 institutions (8.2%) used colonoscopy from the colostomy, 25 institutions (41.0%) used cystoscopy or urethroscopy, 23 institutions (37.7%) used visually confirmation by opening the rectum, 11 institution (18.0%) used insertion of catheter by opening the rectum, 29 institution (47.5%) used only external appearance without opening the rectum and 3 institutions (4.9%) fluorescent ureteral catheter.

Regarding the subgroup analysis in intermediate type of ARMs, cystoscopy or urethroscopy was significantly more frequent in LAARP (70.0%) than in PSARP (25.0%) (OR, 0.17; 95% CI 0.043–0.62, *p* < 0.005). Visually confirmation by opening the rectum was significantly more commonly used in the PSARP group (52.8%) than in the LAARP group (0%) (*p* < 0.001).

### Surgical techniques for closure of RUF in all ARMs and subgroup analysis in intermediate type of ARMs

Table 4 summarizes the results of the questionnaire on surgical techniques for RUF closure.

**Table 4:** Surgical techniques for closure of RUF in all ARMs and subgroup analysis in intermediate type of ARMs

	All ARMs (some overlapping)	Subgroup analysis in intermediate type of ARMs				
		PSARP (*n* = 36) (some overlapping)	LAARP (*n* = 20) (some overlapping)	OR	95% CI	*p* value
What is the main method for closing/ligating the urethral side of the rectourethral fistula? (some overlapping)						
Transfixing suture	39/61 (63.9%)	27/36 (75.0%)	10/20 (50.0%)	2.94	0.81–11.12	0.08
Ligation only	17/61 (27.9%)	8/36 (22.2%)	7/20 (35.0%)	0.54	0.14–2.15	0.35
Clip	4/61 (6.6%)	1/36 (2.8%)	4/20 (20.0%)	0.12	0.002–1.33	< 0.05
Suture closure (interrupted/running)	7/61 (11.5%)	6/36 (16.7%)	0/20 (0%)	Inf	0.69–Inf	0.078
5 mm stapler	3/61 (4.9%)	0/36 (0%)	3/20 (15.0%)	0	0.0–1.28	< 0.05
Nothing (only separation)	0/61 (0%)	0/36 (0%)	0/20 (0%)	–	–	–
What specific materials do you use for closure of the rectourethral fistula? (some overlapping)						
Monofilament absorbable suture	35/61 (57.4%)	21/36 (58.3%)	9/20 (45.0%)	1.69	0.50–5.96	0.41
Braided absorbable suture	24/61 (39.3%)	15/36 (41.7%)	3/20 (15.0%)	3.95	0.90–24.81	0.072
Endoloop	23/61 (37.7%)	13/36 (36.1%)	12/20 (60.0%)	0.38	0.10–1.33	0.10
Metal clip (ligaclip, ligamax)	2/61 (3.3%)	0/36 (0%)	2/20 (10.0%)	0	0.0–2.91	0.12
Polymer clip (hem-o-lok)	2/61 (3.3%)	0/36 (0%)	2/20 (10.0%)	0	0.0–2.91	0.12
Stapler	/613 (4.9%)	0/36 (0%)	3/20 (15.0%)	0	0.0–1.28	< 0.05

*RUF* rectourethral fistula, *ARM* anorectal malformation, *PSARP* posterior sagittal anorectoplasty; *LAARP* laparoscopically assisted anorectoplasty, *OR* odds ratio, *CI* confidence interval

Regarding the main procedure for closing or ligating the urethral side of the RUF in all ARMs, 39 institutions (63.9%) used transfixing sutures, 17 institutions (27.9%) used ligation only, and 4 institutions (6.6%) used clips, 7 institutions (11.5%) used suture closure (interrupted/running), and 3 institutions (4.9%) used 5 mm staplers. No institution reported leaving RUF untreated (only separated).

Regarding the subgroup analysis in intermediate type of ARMs, clips was significantly more frequently used in LAARP (20.0%) than in PSARP (2.8%) (*p* < 0.05) and 5 mm stapler was also significantly more frequently used in LAARP (15.0%) than in PSARP (0%) (*p* < 0.05).

Regarding the specific materials used for fistula closure in all ARMs, 35 institutions (57.4%) used monofilament absorbable sutures, 24 institutions (39.3%) used braided absorbable sutures, 23 institutions (37.7%) used endoloops, 2 institutions (3.3%) used metal clips, two institutions (3.3%) used polymer clips, and 3 institutions (4.9%) used 5 mm stapler.

Regarding the subgroup analysis in intermediate type of ARMs, 5 mm staplers were significantly more frequently used in LAARP (15.0%) than in PSARP (0%) (*p* < 0.05).

## Discussion

The accurate diagnosis of the type of ARM in neonates is crucial for determining the optimal surgical approach. Various imaging modalities have been used, including invertography, cross-table lateral radiography, colostography/fistulography, and ultrasound [8–10]. Invertography is a commonly used traditional method, but it has limitations, including difficulty in positioning the baby upside-down, which causes hypoxia [8]. Colostography/fistulography provides detailed anatomical information but carries the risk of bowel perforation [9]. Ultrasound has emerged as a promising tool for classifying the degree of ARM and visualizing the key structures using various approaches. The pouch-perineum distance, optimally evaluated on the day after birth, can differentiate between low and intermediate/high types of ARMs [11–14]. MRI provides excellent soft tissue delineation of the sphincter muscles and can assist in precise pull-through, similar to MRI-assisted LAARP [10, 15]. However, MRI requires prolonged sedation and may not be easily available. According to the results of the present survey, invertography is still commonly used, whereas cross-table radiography is underutilized. Given the advantages of cross-table radiography over invertography [8], we had assumed that invertography would be performed a little less frequently. Still, it was the most commonly performed diagnostic procedure in the neonatal period. Incorporating ultrasound and selectively using MRI can further improve diagnostic accuracy and assist in the surgical procedure of precise pull-through.

The timing of definitive surgery for ARMs in Japan is relatively late in comparison to other reports [3, 16, 17]. In the present survey, most institutions in Japan performed anorectoplasty at 3–8 months of age, with the majority operating at 5–6 months of age. However, several studies have suggested that early definitive surgery may be beneficial for long-term postoperative bowel function. Performing anorectoplasty in the neonatal period allows for the early establishment of brain-defecation reflexes, potentially improving long-term continence [18, 19]. It also avoids colostomy-related complications and reduces the number of surgeries [18–20]. However, definitive surgery in the neonatal period carries risks of wound infection and dehiscence due to exposure to meconium [19]. With careful patient selection and meticulous surgical techniques, earlier anorectoplasty within the first few months of life may improve the outcome in terms of postoperative bowel function in patients with ARMs.

Regarding surgical procedures for high-/intermediate-type ARM in male patients, LAARP was more frequently performed for high-type ARMs, while PSARP was the preferred approach for intermediate-type ARMs. These findings suggest that surgeons select the operative approach based on the degree of the ARM.

During anorectoplasty for ARMs, the choice of surgical instruments sometimes affects operative outcomes. Bipolar devices are recommended over monopolar or USAD to dissect rectourethral fistulas because bipolar devices provide precise dissection with minimal lateral thermal spread [21]. Blunt dissection, cystoscopy/urethroscopy, and visual confirmation of the fistula by opening the rectum were not significantly different between LAARP and PSARP. Blunt dissection is more commonly used in PSARP, and direct visualization of the fistula is also preferred in PSARP. Cystoscopy/urethroscopy is more frequently used in LAARP to confirm the extent of dissection. These differences highlight the distinct approaches to these techniques. Advances such as fluorescent ureteral catheters may further enhance visualization and safety in LAARP [21]. Ultimately, the choice of instruments should be based on the surgeon’s experience and expertise and the specific case to minimize complications and optimize the maximum outcomes.

Several studies have highlighted the risk of incomplete RUF excision and residual fistula formation after LAARP [5, 6]. A mid-term review demonstrated that intraoperative measurement of the RUF during LAARP can successfully prevent incomplete excision of the RUF [22]. The present survey data showed interesting differences between LAARP and PSARP in the methods used to confirm complete RUF dissection. Cystoscopy/urethroscopy was utilized significantly more frequently in LAARP than in PSARP, likely due to the patient’s position facilitating cystoscopy and the inability to palpate the urethra via the laparoscopic approach, which differs from the approach in PSARP. In contrast, opening the rectum to visually confirm the RUF or inserting a catheter was performed almost exclusively in PSARP, which was enabled by the posterior sagittal approach. The original LAARP description involved fistula ligation without a specific technique to confirm complete excision [2]; however, recent experience and advancements in LAARP have led to procedural modifications to reduce this complication [23]. Standardization and prevalence of adequate treatment for RUF may improve the postoperative outcomes of LAARP.

The treatment of RUF remains a critical aspect in the surgical management of ARMs. Various techniques have been employed with notable differences between LAARP and PSARP. Our present survey revealed that clips and 5 mm staplers were more frequently used for fistula closure in LAARP, while suture closure was the predominant method in PSARP. Interestingly, no cases of fistula division without closure have been reported in either group in Japan. This contrasts with findings from Podevin and Mure, where fistulas were cut without closure in 11 of 34 LAARP cases [24]. The use of clips and staplers in LAARP was a situation-specific approach, with devices that were easier to use in laparoscopic surgery.

## Conclusion

This first nationwide multicenter survey in Japan revealed the current fistula treatment techniques in PSARP and LAARP for intermediate- and high-type ARM. Cystoscopy/urethroscopy was more frequently used to confirm the extent of dissection in LAARP, whereas direct visualization of the fistula was preferred in PSARP. Blunt dissection was more frequent in PSARP, and clips or staplers were used more frequently in LAARP for closure of the RUF than in PSARP. Further studies are required to investigate not only the postoperative bowel function but also the voiding function and sexual function after the application of these fistula treatment methods.

## Data Availability

The data sets generated and/or analyzed during the current study are available from the corresponding author on reasonable request.
